# The effect of paraformaldehyde fixation and PBS storage on the water content of the human lens

**Published:** 2008-01-17

**Authors:** Robert C Augusteyn, Gijs Vrensen, Ben Willekens

**Affiliations:** 1 Vision Cooperative Research Centre, Sydney, Australia; 2 Department of Biochemistry and Molecular Biology, La Trobe University, Bundoora Australia; 3 Department of Ophthalmology, Leiden University Medical Center, Leiden, The Netherlands; 4 Research Team Retinal Signal Processing, Netherlands Institute for Neurosciences, Amsterdam, The Netherlands

## Abstract

**Purpose:**

Fixation and phosphate buffered saline (PBS) storage are frequently used before studies of the morphological, biochemical, and optical properties of the human lens begin. It is assumed that this does not alter the properties being examined. The present study was undertaken to determine the effects of fixation and PBS storage on the human lens wet weight.

**Methods:**

Human donor lenses were incubated in a buffered paraformaldehyde (PF) solution or in PBS and their wet weights were monitored for up to 44 and 13 days, respectively.

**Results:**

PF fixation resulted in a large decrease in wet weight, averaging 25%±2.3% at 30 days for 14 human donor lenses, aged 49–80 years. The loss was essentially complete by 21 days. Out of the 10 lenses, aged 52–71 years, which were incubated in PBS alone, six of them increased in weight by an average of 38% over 13 days and four ruptured within four days. Comparison of literature data for a fixed eight-year-old lens with those for an unfixed seven-year-old lens indicated that the decrease in wet weight was due mainly to a loss of water from the cortex, which resulted in virtual disappearance of the water/protein gradient and the formation of a plateau containing 58% water in over 90% of the lens.

**Conclusions:**

Fixation substantially alters the amount and distribution of water in the human lens. Caution should be exercised when interpreting data on water and protein distributions as well as cell dimensions obtained with lenses which have been fixed. In addition, prolonged storage of a lens in PBS will result in substantial water uptake, which may affect measurements of their dimensions and optical properties.

## Introduction

The ocular lens grows continuously throughout life by mitotic division of the epithelial cells in the anterior pre-equatorial region. The post-mitotic cells subsequently differentiate and elongate to form new cohorts of fiber cells, which cover their predecessors. Unlike in other tissues of ectodermal origin, the ‘older’ cells are not shed and retain the majority of their proteins (crystallins). Instead, they are progressively compacted and form the different parts of the lens nucleus. This compaction is thought to be due to the loss of water. As a consequence, the concentration of protein and, hence, the refractive index gradually increases. This generates a refractive index gradient, increasing from the outside to inside, which is essential for reducing spherical aberration of the lens.

One of the key requirements for understanding the biologic origin of lens optical properties, such as the refractive index, and how these properties change during the development of cataracts and presbyopia is a detailed knowledge of the content and distribution of water and protein in the lens. Several attempts have been made to obtain such information, but most have been complicated by post-mortem changes in the lens and the inability of the techniques used to collect data from discrete locations in the lens. Many of the difficulties appeared to have been overcome through the application of micro-autoradiography of freeze-dried lens slices [1], a microdissection technique [2], and Raman microspectrometry [3,4]. This has yielded detailed data on the distribution of the crystallins and water in human lenses, the changes of these lenses with age [1–5], and on the conformation of the proteins [6].

In a recent re-analysis of the human lens growth using both published and unpublished data [7], it was recognized that the lens wet weights reported by Willekens, Kappelhof, and Vrensen [8] fell well below the weights reported from other laboratories. Therefore, these were not included in the re-analysis. It was noted that these lenses had been fixed in a cacodylate buffered solution of glutaraldehyde and paraformaldehyde as a prerequisite for (ultra)structural and Raman studies referred to above [3–6,8]. It has to be considered that the fixation process might have resulted in a substantial loss of water. If correct, this could have important ramifications for the interpretation of the local protein distribution data such as those obtained in the above Raman studies.

Fixation is commonly used to stabilize the lens before ultrastructural analyses such as studies on the sizes and compaction of fiber cells in different regions of the lens and their changes with age [9–11]. If the fixation and the subsequent embedding procedure had resulted in a significant water loss from these lenses, some of the conclusions about localized compaction and cell sizes may have to be reconsidered.

Therefore, we have examined the effects of paraformaldehyde (PF) fixation and phosphate buffered saline (PBS) storage on human lens weights. Our observations indicate that water loss is indeed considerable during fixation while incubation in PBS results in substantial water uptake.

## Methods

The human eye lenses were obtained from the Cornea Bank of Amsterdam (Dr. E. Pels, Head) after the dissection of corneas for transplantation purposes. The research on this material adhered to the Declaration of Helsinki and is in compliance with the Dutch Law for the use of humans post-mortem and donor material, respectively. The donor eyeballs were collected in the hospital and sent to the eye bank in a moist chamber at a temperature of approximately 4 °C. The time between death of the donor and dissection of the cornea and lens was recorded. This ranged from 4.5 to 36 h. A total of 24 lenses with no visible opacities were collected. For the study of the effect of PF fixation, 14 lenses (six pairs and two single lenses), ranging in age between 61 and 74 years, were dissected, freed from zonular fibers, blotted dry on filter paper, and immediately weighed on a Mettler high precision balance. Subsequently, the lenses were placed in numbered vials containing 1.5% phosphate buffered paraformaldehyde (pH 7.3, 340 mOsm) at room temperature (20 °C). At daily intervals, except on the weekends and a few free days, the lenses were re-weighed after blotting dry on filter paper. This was continued for at least 30 days and at most 44 days for some lenses. For the study of the effect of PBS on lens wet weight, the same procedure was followed. Ten lenses (five pairs), ranging in age between 52 and 71 years, were used. Because of lens ruptures, the PBS measurements were stopped after 13 days.

The water distribution of an unfixed seven-year-old lens was calculated from the published refractive index (RI) distribution [12] by first calculating the protein concentration using refractive increments as determined by Pierscionek, Smith, and Augusteyn [13] and then calculating the water content using a value of 0.74 for the partial specific volume of the proteins. This was compared with the water distribution of an eight-year-old lens as measured using Raman microspectroscopy [5].

Lens diameter and axial thickness values obtained from published in vivo [14] and in vitro [8,15,16] measurements were used to calculate lens volumes, using the equation for an ellipsoid, assuming perfect rotational symmetry about the optical axis. This may overestimate volumes by a maximum of 5% [15].

## Results

**Figure 1:**
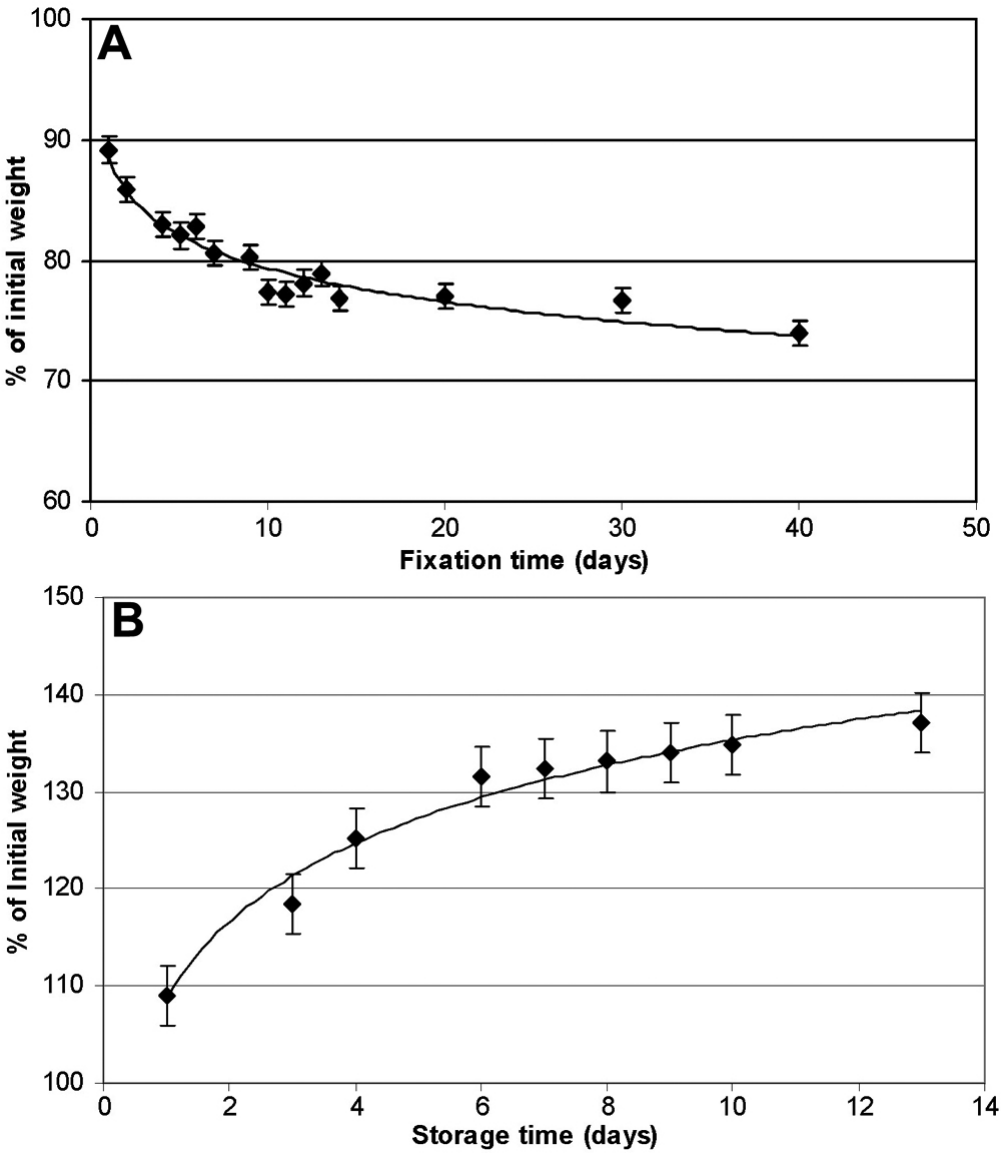
The effect of paraformaldehyde fixation and incubation in phosphate buffered saline on the wet weights of human lenses. The effects of fixation (**A**) were examined by incubating freshly excised human lenses at room temperature in 1.5% formalin in PBS up to a maximum of 44 days. The lenses were removed at regular intervals, blotted dry, and weighed before being replaced in the fixative. The data shown in (**A**) are the averages for 14 lenses, aged 61–74 years. The data for the effects of incubation in PBS alone (**B**) are the averages from six freshly excised human lenses, aged 52–71.

Fourteen human lenses, ranging in age from 61 to 74 years, were placed in the fixative solution (1.5% PF in PBS), and their weights were monitored for up to 44 days (Figure 1A).

Lens weight decreased rapidly with over 12% lost in the first day. Thereafter, the rate gradually decreased, and the weight loss was virtually complete around 21 days. The other 10 lenses, placed in PBS only, served as controls (Figure 1B). In contrast to the lenses in a fixative, these rapidly took up water. Six of the lenses increased in weight by almost 40% over 13 days and appeared to be still slowly accumulating water. The remaining four lenses ruptured within four days.

**Figure 2:**
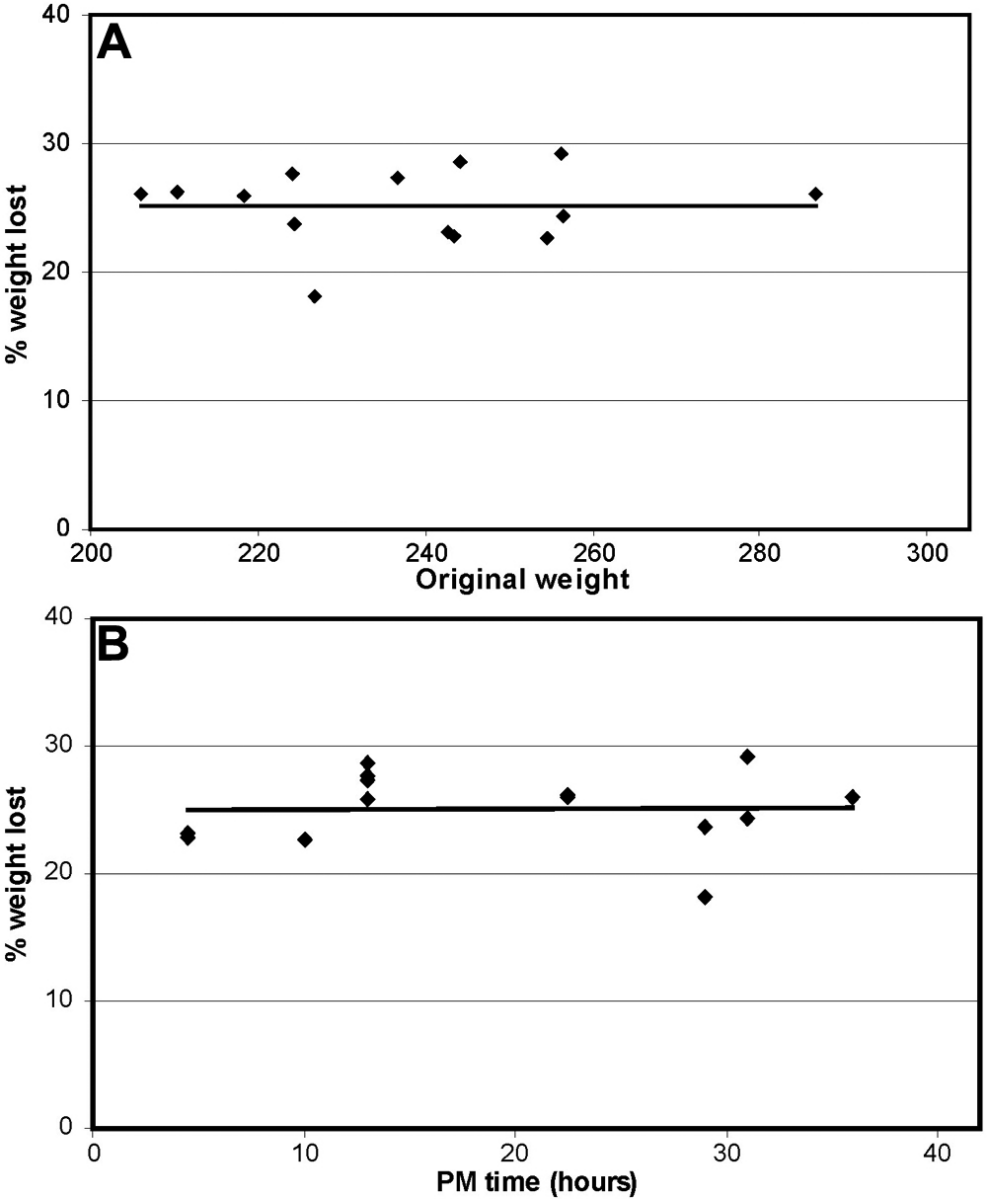
Relationship between maximum weight loss during fixation and initial lens weight and post-mortem time. Human lenses, ranging in age from 61 to 74 years, were incubated for up to 44 h in 1.5% paraformaldehyde in PBS and the maximum amount of weight lost was compared with the initial lens wet weight (**A**) and with the post-mortem (PM) time (**B**). No trends can be observed for either plot and the average weight loss is 25%±2.3%.

As can be seen in Figure 2, the weight loss did not appear to be related to either the initial lens weight, which ranged from 206 to 287 mg, or to the post-mortem time which ranged from 6 to 36 h. There was also no relationship with the age of the donor over the range examined (49–80 years old). For all lenses, the water loss averaged 25%±2.3% of the original lens weights. Similarly, no relationship was found for the water uptake by lenses placed in PBS.

## Discussion

The present study was undertaken because of a recent report [7] that the wet weights of fixed lenses described by Willekens et al. [8] were substantially lower than those of unfixed lenses described by other laboratories. No significant differences have been observed between the dry weights of fixed and unfixed lenses of the same age indicating that no solids are lost from the lens during fixation (Augusteyn, unpublished observations). Therefore, the data presented here show clearly that the low weight can be attributed to the substantial (25%) loss of water during fixation.

Willekens et al. [8] determined lens weights after seven days of fixation at which time a 19.7% decrease in weight would have occurred according to our current measurements. Correcting the previously published data for this loss yields values for wet weights statistically indistinguishable from unfixed lenses freshly collected for the present study and from lenses used in the recent re-analysis of lens growth [7].

**Figure 3:**
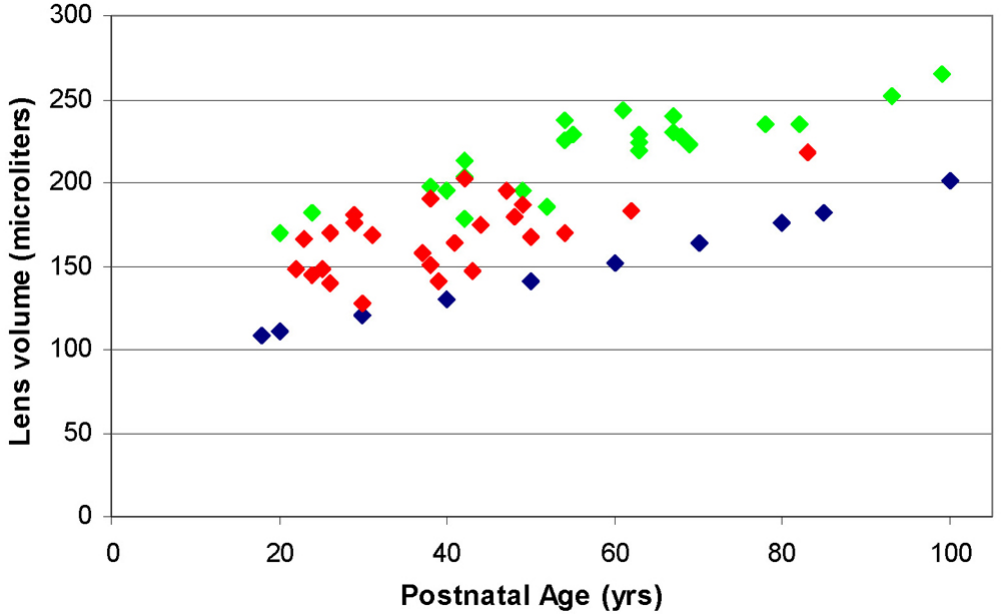
Comparison of the volumes of fixed and unfixed lenses. The volumes of fixed (blue diamond) [8] and unfixed (green diamond) [12] in vitro lenses and fully accommodated in vivo lenses (red diamond) [14] are shown. Volumes of unfixed lenses were calculated using the published dimensions for individual lenses, assuming an ellipsoid shape. Fixed lens volumes were calculated using values calculated from the published regression equations for thickness and diameter [8].

These observations suggest that there could be substantial decreases in the dimensions and volume of the lens on fixation. This was examined by comparing the published data for fixed lens dimensions with those of unfixed lenses and calculating volumes from these. From the best fit of the data presented by Willekens et al. [8], fixed lens thickness ranged around 3–4 mm between the ages of 20 and 80 years. This may be contrasted with the 4.2–5 mm reported for unfixed lenses [15,16] and 4–5 mm for the fully accommodated in vivo lenses [14] over the same age range. To avoid errors due to shape changes in the lenses, volumes were calculated where dimensions were available. Figure 3 shows the volume comparisons for fixed, unfixed, and in vivo lenses.

Although there is some variability in the data, it would appear that the volumes of unfixed in vitro and in vivo lenses are comparable, ranging between 150 and 230 µl over the 20–80 year age range. Any difference can probably be attributed to the uptake of water by the post-mortem lens [17]. By contrast, the fixed lenses are substantially smaller at 110–176 µl, almost exactly 25% lower, in close agreement with the observed weight loss in the present study.

**Figure 4:**
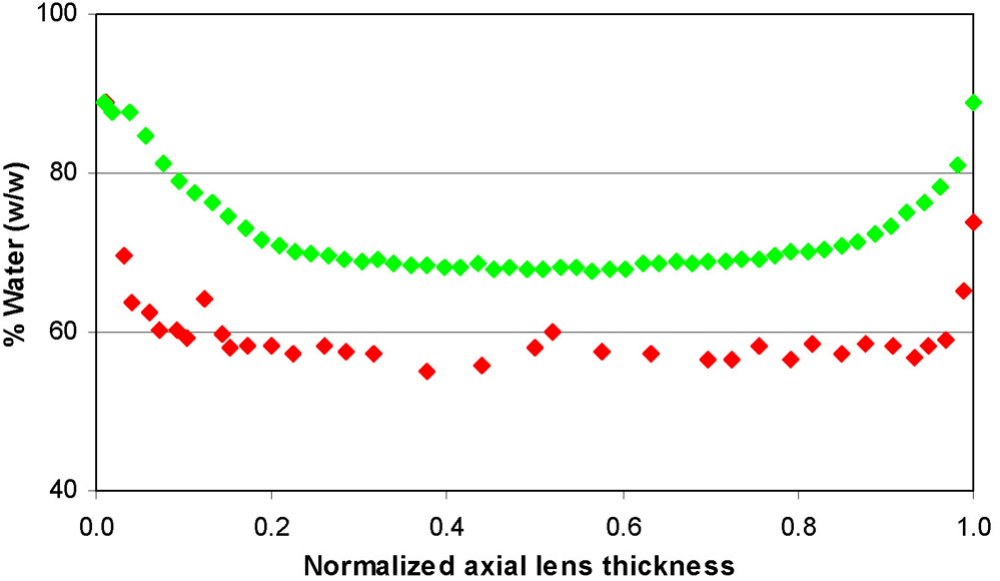
The distribution of water along the sagittal axis in fixed and unfixed human lenses. Comparison of the distributions of water along the sagittal axis in an unfixed seven-year-old lens (green diamond), which was calculated from the RI gradient obtained with MRI [12], and in a fixed eight-year-old lens (red diamond) as determined by the use of Raman microspectroscopy [5] is shown. The dimensions were normalized to eliminate differences in the thickness of the lenses due to possible shrinkage of the fixed lens.

To determine from where the water was lost, the water distribution in a fixed eight-year-old lens described by Siebinga et al. [5] was compared with that calculated from the refractive index gradient observed in an unfixed seven-year-old lens [12]. The distributions along the sagittal (optical) axes are shown in Figure 4. Essentially the same differences were found for the equatorial axis.

The fixed lens had lower water contents almost along the whole axis. Between 0.06 and 0.97 of the normalized distance, the water content in the fixed lens averaged 58% (w/w). In the unfixed lens, the lowest water content was around 68%, but this was limited to the central 0.4–0.6 of the normalized distance. Much larger differences are observed in the periphery. As can be seen from Figure 4, the outer 0.2 of the normalized axis contains the water/refractive index gradient in the unfixed lens. Following fixation, this region has become much narrower (0.03–0.06) with the virtual disappearance of the gradient. In terms of actual dimensions in the sagittal plane of the eight-year-old fixed lens, the width of the gradient had reduced from the unfixed 1.2 mm [12,18] to an average of 0.2 mm while in the equatorial plane it decreased from 1.2 mm to 0.6 mm.

Calculation of the total water content in the inner region (from 0.2 to 0.8 along the normalized axes) and the outer (from 0 to 0.2 plus from 0.8 to 1.0 of the normalized axes) revealed that >90% of the water lost probably came from the cortex.

It was also reported from the Raman measurements that the nuclear water content increases with age from 58% to near 70% around the age of 80 [5]. This is inconsistent with the trends in protein concentrations determined by microautoradiography [1] and microdissection [2], with the water content determined using thermogravimetric analysis [19], and with the development of a plateau of refractive index observed with MRI [18]. The reason for this apparent increase with the fixed lenses is not obvious, but it seems unlikely that the normal nucleus could accommodate such a large influx of water without an obvious disruption of its ordered structure. The likely explanation of this observation is that there is an age-related decrease in the amount of water that could be lost from the lens center during fixation.

Several ultrastructural studies on lens cell sizes in different regions of the lens at different ages have been conducted with fixed lenses [9–11]. It is not possible to assess how much water was lost since dimensions were not reported for the fixed tissues. Our data suggest that water loss from the lens during fixation is not uniform and, as suggested by the Raman observations, may even vary with age. How this might affect cell dimensions in lens layers, such as the embryonic and the fetal, juvenile, and adult nuclei as well as different regions of the cortex, cannot be gauged without further information. However, the possibility of significant change cannot be ruled out.

At first, the loss of water during fixation appears rather surprising given that the osmolality of the fixing solution is essentially the same as that of PBS, which resulted in water uptake. The lowest water content observed in the fixed seven-year-old lens was around 58%, and this encompassed almost all of the lens [5]. This suggests that free water is lost and compaction of human cells may be limited to this final concentration of water because the residual water is tightly bound to the crystallins and can only be removed by more forcing conditions such as heating or vacuum drying. However, it is not clear what the driving force for such water loss may be. More likely, it may not be the free water which is lost.

Fixation of the lens proteins modifies hydrophilic side chains and generates crosslinked aggregates. This would reduce their osmolality and hydration shells with a concomitant release of water, which would move out of the lens. In the nucleus, the proteins have already been substantially modified through age-related processes [reviewed in 20] so there is less water loss from this region. It can be calculated from the data presented by Bettelheim and coworkers [21] that bound (nonfreezable) water in a 50-year-old lens represents around 25% of the lens weight, remarkably close to the weight loss observed in the current study. This suggests that the water lost from the lens may actually have come from the bound fraction in the crystallins.

Storage in PBS leads to a significant increase in wet weight and even to the rupture of some lenses. The observation that lenses swell when placed in an apparently isotonic medium is not new. Uptake of water and/or an increase in lens thickness have frequently been observed in lenses left in the eye for prolonged periods or in culture media and salt solutions [17,22,23]. This can probably be attributed to a failure of the volume-regulating systems due to a lack of nutrients. It was noted by Augusteyn et al. [17] that lens swelling along the optic axis and capsular separation could often be detected within hours of a lens being placed in simple salt solutions. Incubation in nutrient-rich media such as DMEM or TC199 reduced the incidence of lens swelling but did not prevent it completely. Schachar found lens thickness started to increase within two days of storage in Optisol, leading to a 10% increase in lens thickness in 10 days [23]. Such increases could have serious implications for in vivo studies on lens optical and physical properties.

Although systematic studies have not been undertaken on other species, it has been observed in our laboratory that baboon, cow, dunnart, kangaroo, rabbit, rhesus monkey, sheep, and toad lenses also shrink when fixed in paraformaldehyde. Incubation of cow, cynomolgus monkey, kangaroo, rhesus monkey, rat, and sheep lenses in PBS results in swelling. This suggests that the phenomena described for human lenses may be universal.

The observations presented here indicate that caution needs to be exercised when interpreting data obtained from lenses, which have been fixed or stored in PBS. This would include data on the distribution of water [3–5] as well as data on protein conformation [6] and on fiber cell dimensions [9–11]. It would appear that the properties of the lens, and especially the cortex, have been grossly altered by fixation so none of the data should be considered as representative of the native in vivo state.

## References

[1] Fagerholm PP, Philipson BT, Lindstrom B (1981). Normal human lens – the distribution of protein.. Exp Eye Res.

[2] Bours J, Wegener A, Hofmann D, Fodisch HJ, Hockwin O (1990). Protein profiles of microsections of the fetal ands adult lens during development and ageing.. Mech Ageing Dev.

[3] Huizinga A, Bot AC, de Mul FF, Vrensen GF, Greve J (1989). Local variations in absolute water content of human and rabbit eye lenses measured by Raman microspectroscopy.. Exp Eye Res.

[4] Bot ACC, Huizinga A, de Mul FFM, Vrensen GJM, Greve J (1989). Raman microspectroscopy of fixed human and rabbit eye lenses and lens slices; new potentialities.. Exp Eye Res.

[5] Siebinga I, Vrensen GF, De Mul FF, Greve J (1991). Age-related changes in local water and protein content of human eye lenses measured by Raman microspectroscopy.. Exp Eye Res.

[6] Siebinga I, Vrensen GF, Otto K, Puppels GJ, De Mul FF, Greve J (1992). Ageing and changes in protein conformation in the human lens: a Raman microspectroscopic study.. Exp Eye Res.

[7] Augusteyn RC (2007). Growth of the human eye lens.. Mol Vis.

[8] Willekens B, Kappelhof J, Vrensen G (1987). Morphology of the aging human lens.. Lens Res.

[9] Taylor VL, al-Ghoul KJ, Lane CW, Davis VA, Kuszak JR, Costello MJ (1996). Morphology of the normal human lens.. Invest Ophthalmol Vis Sci.

[10] Al-Ghoul KJ, Costello MJ (1997). Light microscopic variation of fiber cell size, shape and ordering in the equatorial plane in bovine and human lenses.. Mol Vis.

[11] Al-Ghoul KJ, Nordgren RK, Kuszak AJ, Freel CD, Costello MJ, Kuszak JR (2001). Structural evidence of human nuclear fiber compaction as a function of ageing and cataractogenesis.. Exp Eye Res.

[12] Jones CE, Atchison DA, Meder R, Pope JM (2005). Refractive index distribution and optical properties of the isolated human lens measured using magnetic resonance imaging (MRI).. Vision Res.

[13] Pierscionek B, Smith G, Augusteyn RC (1987). The refractive increments of bovine α-, β- and γ-crystallins.. Vision Res.

[14] Strenk SA, Semmlow JL, Strenk LM, Munoz P (1999). Groniund-Jacob Jm DeMarco JK. Age-related changes in human ciliary muscle and lens: A magnetic resonance imaging study.. Invest Ophthalmol Vis Sci.

[15] Rosen AM, Denham DB, Fernandez V, Borja D, Ho A, Manns F, Parel JM, Augusteyn RC (2006). In vitro dimensions and curvatures of human lenses.. Vision Res.

[16] Schachar RA (2005). Growth patterns of fresh human crystalline lenses measured by in vitro photographic biometry.. J Anat.

[17] Augusteyn RC, Rosen AM, Borja D, Ziebarth NM, Parel JM (2006). Biometry of primate lenses during immersion in preservation media.. Mol Vis.

[18] Augusteyn RC, Jones C, Pope JM (2008). Development of a refractive index plateau in the nucleus of the human lens.. Clin Exp Optom.

[19] Heys KR, Cram SL, Truscott RJW (2004). Massive increase in stiffness of the human lens nucleus with age: the basis for presbyopia?. Mol Vis.

[20] Augusteyn RC, Stevens A (1998). Macromolecular structure of the eye lens.. Prog Polym Sci.

[21] Lahm D, Lee LK, Bettleheim FA (1985). Age dependence of freezable and nonfreezable water content of normal human lenses.. Invest Ophthalmol Vis Sci.

[22] Augusteyn RC, Cake MA (2005). Post mortem uptake of water by sheep lenses left in the eye.. Mol Vis.

[23] Schachar RA (2004). Central surface curvature of postmortem-extracted intact human crystalline lenses.. Ophthalmology.

